# A review of model evaluation metrics for machine learning in genetics and genomics

**DOI:** 10.3389/fbinf.2024.1457619

**Published:** 2024-09-10

**Authors:** Catriona Miller, Theo Portlock, Denis M. Nyaga, Justin M. O’Sullivan

**Affiliations:** ^1^ The Liggins Institute, The University of Auckland, Auckland, New Zealand; ^2^ The Maurice Wilkins Centre, The University of Auckland, Auckland, New Zealand; ^3^ MRC Lifecourse Epidemiology Unit, University of Southampton, Southampton, United Kingdom; ^4^ Singapore Institute for Clinical Sciences, Agency for Science Technology and Research, Singapore, Singapore

**Keywords:** metrics, machine learning, genomics prediction, clustering, classification, regression, disease prediction

## Abstract

Machine learning (ML) has shown great promise in genetics and genomics where large and complex datasets have the potential to provide insight into many aspects of disease risk, pathogenesis of genetic disorders, and prediction of health and wellbeing. However, with this possibility there is a responsibility to exercise caution against biases and inflation of results that can have harmful unintended impacts. Therefore, researchers must understand the metrics used to evaluate ML models which can influence the critical interpretation of results. In this review we provide an overview of ML metrics for clustering, classification, and regression and highlight the advantages and disadvantages of each. We also detail common pitfalls that occur during model evaluation. Finally, we provide examples of how researchers can assess and utilise the results of ML models, specifically from a genomics perspective.

## 1 Introduction

The general hype around the generative artificial intelligence (AI) era has increased the popularity of machine learning (ML) for a range of applications. Alongside this, the advent of “plug and play” style ML tools, such as PyCaret, has dramatically increased the accessibility of ML to scientists and researchers without a traditional computational background (Ali, 2020; Manduchi et al., 2022; Whig et al., 2023). In genomics, ML is becoming increasingly used to analyse large and complex datasets, including sequencing data (Caudai et al., 2021; Chafai et al., 2024). Therefore, it is increasingly important that “all” researchers understand what happens after an ML model has been deployed. This is particularly true for the choice of performance metrics and how to interpret the validity of the results. As such, without understanding the common metrics used in ML, together with an awareness of the inherent strengths and weaknesses of such metrics, there is a possible risk of result inflation (Kapoor and Narayanan, 2023). Therefore, understanding the potential biases within the input data is essential to successfully interpret the results (Vokinger et al., 2021).

Existing reviews of ML applications to genetic and genomic datasets either focus on earlier stages of the ML pipeline (e.g., feature selection, method selection), or give an overview of the whole process (Libbrecht and Noble, 2015; Ho et al., 2019; Musolf et al., 2022; Pudjihartono et al., 2022). This review addresses an important gap in the literature by focusing on the final section of the ML pipeline – model evaluation. Specifically, we cover the most common use cases of ML in genomics before an in-depth analysis of the metrics used to evaluate each subtype, including the advantages and disadvantages of each. We finalise by cautioning researchers and scientists of the common pitfalls that can bias model performance and inflate the metrics reported.

### 1.1 Types of ML typically used in genomics

**FIGURE 1:**
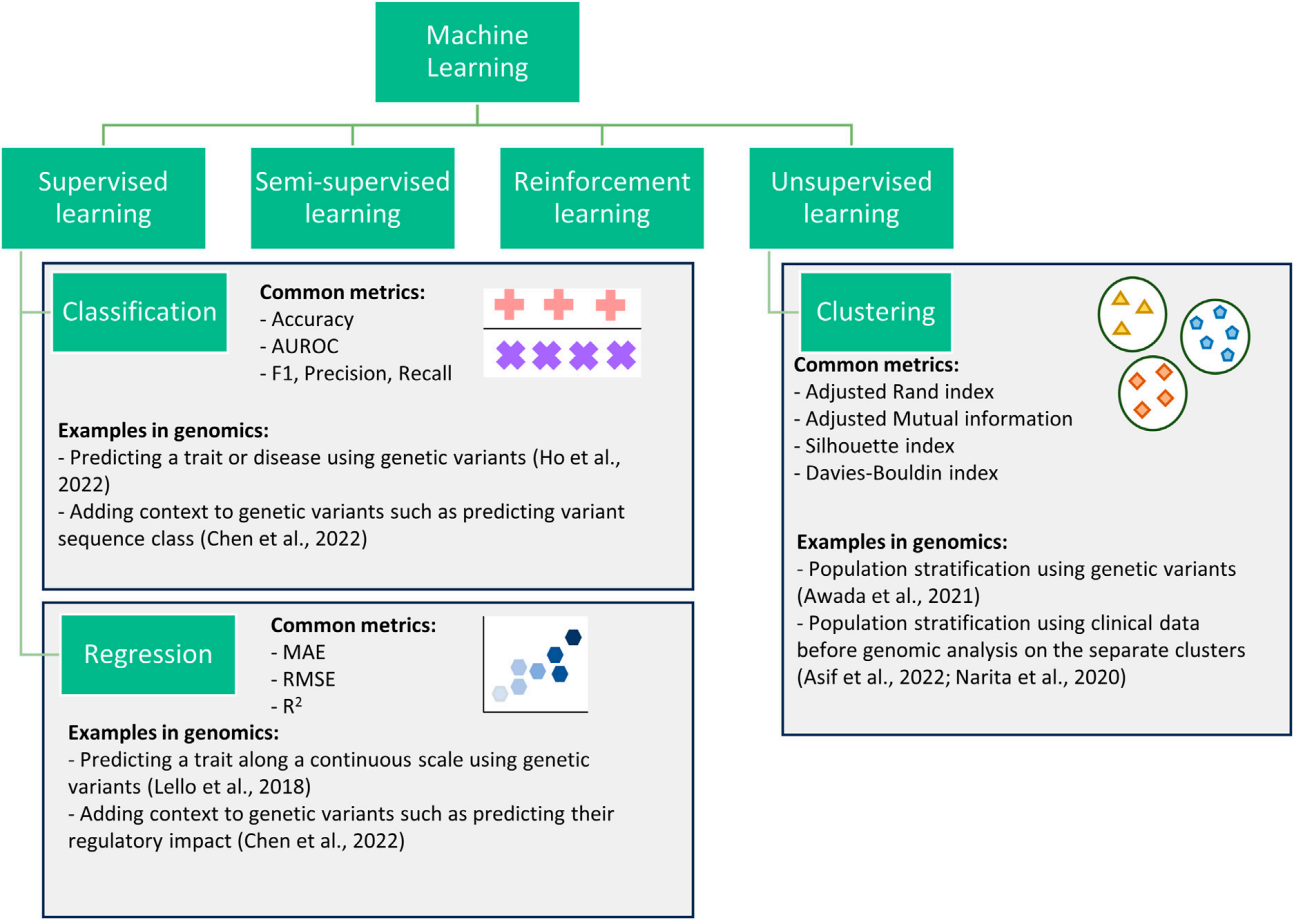
Flowchart showing four categories of machine learning. This review focuses on three subcategories (classification, regression, and clustering) within the supervised and unsupervised categories.

Supervised learning, unsupervised learning, semi-supervised learning, and reinforcement learning are the four main types of ML algorithms used within genetic and genomic datasets (Figure 1) (Libbrecht and Noble, 2015; Ho et al., 2019; Koumakis, 2020; Bracher-Smith et al., 2021). Here we focus on unsupervised and supervised learning and their subcategories: clustering (unsupervised learning), classification and regression (supervised learning).

Clustering algorithms are processes for the identification of subgroups within a population. Clustering can be performed when data is prelabelled (e.g., known disease subtypes) or with no a priori information. Clustering has been successfully used to improve prediction (Alyousef et al., 2018), to identify disease-related gene clusters (Di Giovanni et al., 2023), or to better define complex traits/diseases (Lottaz et al., 2007; Lopez et al., 2018; Yin et al., 2018; Awada et al., 2021).

Classification algorithms encompass all machine learning methods where pre-labelled data is used to train an algorithm to predict the correct class, where class refers to all data points with a given label (e.g., control class or a specific disease class). These are commonly used within genomics to predict a trait/disease (i.e., diagnostics) (Trakadis et al., 2019; Lee and Lee, 2020; Ho et al., 2022), or to identify potential biomarkers (Al-Tashi et al., 2023). However, they can struggle with imbalanced datasets, where one class is significantly more prevalent than the other, leading to biased predictions (Ramyachitra and Manikandan, 2014).

Regression algorithms, like classification algorithms, predict a target variable for each datapoint or individual; however, they are applied in applications involving continuous variables. For example, regression algorithms are commonly used for the prediction of highly heterogeneous traits with known scales such as height, systolic blood pressure, and waist-hip ratio (Bellot et al., 2018; Lello et al., 2018). While regression algorithms can capture complex relationships between variables, they are sensitive to outliers which can impact the reliability of predictions (Wang, 2021). This review only covers regression for continuous variables. Other methods, such as negative binomial and Poisson used in mutation burden analysis and differential gene expression analysis (Sun et al., 2017; Li et al., 2019; Zhang et al., 2020), are outside of the scope.

Classification and regression algorithms have also been applied to add context to genomic data such as predicting the regulatory impacts of single nucleotide polymorphisms (SNPs). For example, the interpretable deep learning sequence model Sei predicts sequence regulatory activity based on chromatin profiles (Chen et al., 2022). Such a framework can be considered both classification and regression as it predicts a variant’s sequence class (classification) and provides a regulatory impact score (regression). In this case, classification provides users with a more understandable output (e.g., promoter) but loses some of the information, whereas the regression score captures more information but is less interpretable. Therefore, by providing both a classification and regression output, users can decide between increased interpretability and information.

Clustering, classification, and regression algorithms all have multiple metrics for evaluating their performance and this review focuses on the most commonly used ones in genomics (Figure 1). This review focuses on their applicability for evaluating models in the fields of genetics and genomics. However, the majority of the metrics detailed are also used for hyperparameter tuning during cross-validation. The choice of the metric for tuning can greatly impact the model produced, often resulting in a model that scores highly for the provided metric at the expense of the other metrics. Therefore, the advantages and disadvantages (both general and genomic specific) discussed for each metric in this review are still largely relevant when choosing a metric for hyperparameter tuning. Yang and Shami (2020) provides a review of hyperparameter optimisation.

## 2 ML metrics for clustering

**FIGURE 2:**
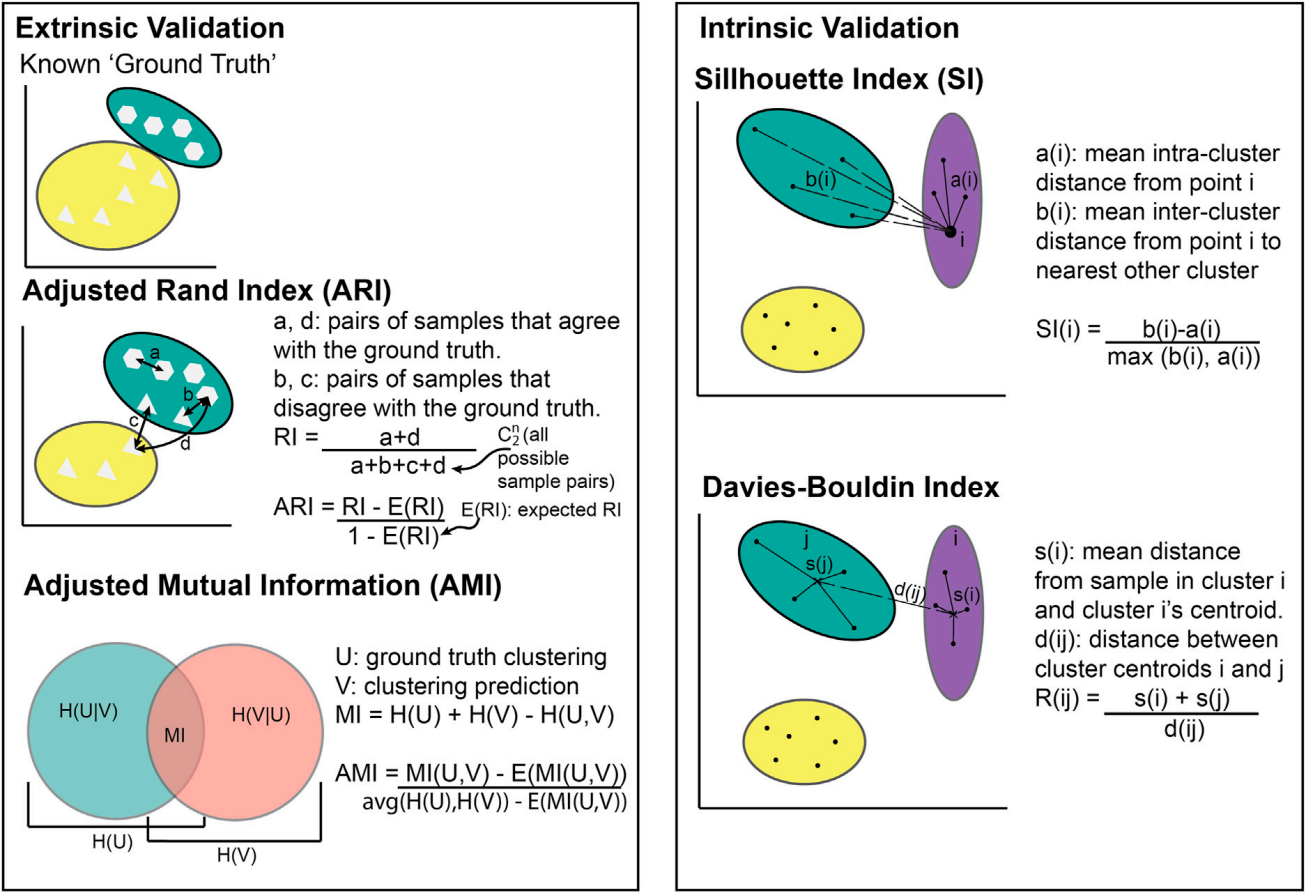
Illustration of cluster metric calculations. Extrinsic validation methods require known clusters to compare against whilst intrinsic validation does not.

The choice of metric for evaluating clustering algorithms largely depends on whether there is access to a “ground truth” (Box 1). If there are known categories to compare the clusters to, extrinsic measures can be used such as the Adjusted Rand Index (Hubert and Arabie, 1985) or Mutual Information (Vinh et al., 2010) (Figure 2). Without a ground truth, intrinsic measures must be used (e.g., the Sillhouette index or Davies Bouldin index). Intrinsic metrics measure the similarities between points within the same cluster compared to the similarity between clusters (Figure 2). They score highly if the intra-cluster similarity is greater than the inter-cluster similarity. Extrinsic metrics score highly if the clusters are similar to the known ground truth clusters (Figure 2).

BOX 1 **| Glossary**

Class – a group of samples or individuals with the same target variable. For example, a control and asthmatic would be two classes in a classification analysis.

Clustering ground truth – a known set of clusters for a given dataset.

Decision boundary – a score threshold used in classification algorithms to assign individuals to classes.

Euclidean distance – the length of the line segment that would connect two points.

Imbalanced dataset – a dataset where one class(es) appears at a much higher rate than the other class(es).

Intra-cluster similarity – similarity between datapoints assigned to the same cluster.

Inter-cluster similarity – similarity between datapoints assigned to different clusters.

True positive rate (TPR) – also known as recall. The percentage of “positive” samples that have been correctly labelled as “positive”.

False positive rate (FPR) – the percentage of ‘negative’ samples that are incorrectly classified as “positive”.

### 2.1 Adjusted Rand Index

The Adjusted Rand Index (ARI) is a measure of similarity between two clusterings of the same dataset, while accounting for similarities that occur by chance (Hubert and Arabie, 1985). For example, the ARI can be used to compare the similarity between calculated clusters within a disease group and known clusters based on disease subtypes.

$$ARI=\frac{RI-E}{1-E}\;where\;RI=\frac{a+d}{C_{2}^{n}}\;and\;E=\frac{\sum \left(C_{2}^{n_{i}}\right)\times \sum \left(C_{2}^{n_{j}}\right)}{C_{2}^{n}}$$

Given:

- n = number of samples in the dataset
- a and d = number of pairs of samples in the same and different clusters between the two clusterings respectively
- n_i_ and n_j_ = number of samples in clusters i and j respectively

If ARI = −1, it indicates complete disagreement (i.e., no individuals are in the same cluster as the known ground truth), while ARI = 0 indicates an agreement equivalent to that from random chance, and ARI = 1 indicates perfect agreement (i.e., all individuals are in the same cluster as the known ground truth). Figure 2 shows most individuals placed in the same cluster as the known ground truth, meaning the ARI would be between 0 and 1.

ARI is a common metric choice for validating the performance of a clustering technique within biology (Shi et al., 2022; Zhen et al., 2022). However, ARI is based on the assumption that the known clusters are correct for the use case. For example, if the aim of clustering is to identify novel groups within a population (diseased or otherwise) or to identify similarities between genetic variants, comparing against known clusters would be detrimental to the problem (Awada et al., 2021). Another limitation is ARI’s bias to cluster size. If a clustering contains a mixture of large and small sized clusters, ARI will be predominantly influenced by the large clusters (Warrens and van der Hoef, 2022).

### 2.2 Adjusted Mutual Information

Adjusted Mutual Information (AMI) is a clustering metric that comes from information theory (Vinh et al., 2010). It calculates how much information is shared between two clusterings (i.e., known clusters and calculated clusters).

$$AMI\left(U,V\right)=\frac{MI\left(U,V\right)-E\left(MI\left(U,V\right)\right)}{avg\left(H\left(U\right),H\left(V\right)\right)-E\left(MI\left(U,V\right)\right)}$$

- U and V = two clusterings (e.g., calculated clusters and known clusters)
- H = individual entropy – a measure of expected uncertainty
- MI = mutual information algorithm described by Vinh et al. (2010).
- E = the expected value based on chance.

Both AMI and ARI adjust for chance and can be used to calculate an algorithm’s performance when a “ground truth” is known. Therefore, deciding when it is appropriate to prioritize one metric over the other can be difficult. The key differentiating factor derives from the fact that ARI scores solutions with similar sized clusters higher. By contrast, AMI is biased towards “pure” clusters, consisting of only one class type and are often imbalanced (Romano et al., 2016). For example, if some disease subtypes are rarer than others resulting in imbalanced cluster sizes, AMI is likely to be a more accurate metric than ARI. Variations of AMI measures have been used in biology, including to create gene regulatory networks (Shachaf et al., 2023), identify SNP interactions (Cao et al., 2018), and to analyse similarities between biomarkers (Keup et al., 2021).

### 2.3 Silhouette index

The Silhouette Index (SI) is a common metric that is typically used when there are no labels for the data being clustered. It compares the similarity within a cluster to the similarity between clusters (Rousseeuw, 1987).

$$SI=\frac{1}{N}\sum s\left(i\right)\;where\;s\left(i\right)=\frac{b\left(i\right)-a\left(i\right)}{\left.\max\;\left\{a\right.\left(i\right),b\left(i\right)\right\}}$$

Given:

- N = number of samples in the dataset
- a(i) = mean within cluster distance for sample i
- b(i) = mean distance between sample i and samples within the nearest cluster

SI values range from −1 to 1 with negative values indicating that the average sample has been assigned to “the wrong cluster.” Higher scores (approaching 1) indicate robust clustering and the presence of dense, well-separated clusters. In biological use cases, stratifying individuals can be nuanced meaning clusters could be weaker. As such, there is no guideline for an SI value that acts as a cut-off for “good” clustering for biological data. Rather, the SI threshold varies between use cases (Pagnuco et al., 2017; Zhao et al., 2018).

The SI metric does not rely on labels or measure prediction validity. Therefore, the SI metric is helpful for evaluating the comparative performances of different clustering methods. However, the SI metric cannot detect if the clustering is due to a bias in the data that is unrelated to the trait (Chhabra et al., 2021). For example, when clustering whole genome sequencing data, the clusters may be related to ancestry, sex, or other traits distributed across the population and not the actual trait being studied. Another disadvantage is the key assumption that clusters are Gaussian, meaning that any SI values for data that does not follow a spherical shape will be misleading (Thrun, 2018). For example, if a disease has a limited number of genes associated with it, the genes would not cover enough dimensions to be spherical and satisfy this assumption. Sparsity can also result in irregular shapes. Therefore, the SI metric would not be suitable in some cases, such as rare disease clustering, and should always be used with caution. Nonetheless, it is a useful method in genetics and genomics where clusters are often unknown so there are no labels to compare against (Lopez et al., 2018; Yin et al., 2018).

### 2.4 Davies-Bouldin Index

A less common intrinsic method for evaluating clustering performance is the Davies-Bouldin Index (DBI). This metric compares the similarity between each cluster and the cluster most similar to it (Davies and Bouldin, 1979).

$$DBI=\frac{1}{N}\sum_{i=1}^{N}\max\;\left(R_{ij}\right)\;where\;R_{ij}=\frac{s_{i}+s_{j}}{d_{ij}}\;and\;i\neq j$$

Given:

- N = number of clusters
- s_i_ = the mean distance between each sample in cluster i and cluster i’s centroid
- d_ij_ = the distance between cluster centroids i and j

DBI is an intrinsic method and shares many advantages and disadvantages with the SI. However, unlike the SI, the lower the DBI, the better the samples are clustered with zero being the minimum score. The computation of the DBI is simpler and more efficient than for the SI (Petrovi’c, 2006). This is a particularly valid consideration for the analysis of large genomics datasets, particularly if the data being clustered is whole-genome sequencing data. A limitation of DBI is that the clustering algorithm for its generation requires the Euclidean distance between cluster centroids (Davies and Bouldin, 1979). This is typically not a problem for genomics as Euclidean distance is a common choice in bioinformatic analyses. However, different distance matrices can provide different, even conflicting results and there are times when another distance measure may be more suitable for the research question (Jaskowiak et al., 2014). For example, genomics datasets such as whole genome sequencing data often suffer from sparsity meaning that most of the data is zeroes (Yazdani et al., 2015). In these cases, DBI would not be suitable.

### 2.5 Other clustering metrics

While these four clustering metrics cover the majority of use cases within genomics, there are other metrics that have their advantages. These include internal metrics such as the Calinski-Harabasz index (variance ratio criterion) (Caliñski and Harabasz, 1974; Babichev et al., 2017; Huang et al., 2021) as well as external metrics such as the Fowlkes-Mallows index (Fowlkes and Mallows, 1983; Ryšavý and Železný, 2017; Lee et al., 2023). Methods such as gap statistics are predominantly used for selecting the number of clusters, however, can be used as a metric (Tibshirani et al., 2001; Lugner et al., 2021). Advantages and disadvantages as well as previous uses of these are included in Table 1.

**TABLE 1:** Overview of the common clustering, classification, and regression metrics including their advantages, disadvantages, and example uses in genetics and genomics.

Metric name	Description	Advantages	Disadvantages	References
Adjusted Rand Index (ARI)	Compare similarity between calculated clusters and a ground truth (or different clustering) (Hubert and Arabie, 1985). For example, predicted clusters in a disease group and known disease subtypes	- Compared to Rand Index, corrects for when the number or size of clusters could be impacted by chance (Hubert and Arabie, 1985). Important for genetics where there is high dimensionality - No bias toward certain cluster shapes (Steinley, 2004)	- Requires a known ground truth clustering set so cannot be used if you want to identify new variant or disease subtypes - Biased to cluster size, influenced by large clusters (Warrens and van der Hoef, 2022) - Not applicable to overlapping clusters (e.g., genes in multiple pathways in pathway analysis)	- Clustering of microbiome data (Shi et al., 2022) - Clustering of single-cell Hi-C data (Zhen et al., 2022) - Clustering differentially expressed cancer genes (Wang et al., 2022)
Adjusted Mutual Information (AMI)	Compare similarity between calculated clusters and a ground truth (or different clustering) (Vinh et al., 2010). Similar to ARI, but more suitable for rare disease subtypes (i.e., imbalanced clusters)	- Biased towards pure clusters, not dependent on cluster size. More suitable for imbalanced clusters (e.g., rare diseases) (Romano et al., 2016)	- Requires a known ground truth clustering set so cannot be used if you want to identify new variant or disease subtypes	- Creating gene regulatory networks (Shachaf et al., 2023) - Identifying genetic variant interactions (Cao et al., 2018) - Analyse biomarker similarities (Keup et al., 2021)
Fowlkes-Mallows Index	Compare similarity between calculated clusters and a ground truth (or different clustering). The geometric mean of precision and recall for the clustering (Fowlkes and Mallows, 1983)	- No bias toward certain cluster shapes so can compare different clustering algorithms (Fowlkes and Mallows, 1983)	- The index is biased toward a small number of clusters (Wagner and Wagner, 2007)	- Estimating the sequence similarity of two genomes (Ryšavý and Železný, 2017) - Creating genetic similarity matrices for population substructures (Lee et al., 2023)
Silhouette Index (SI)	Compares the similarity within clusters to the similarity between clusters (Rousseeuw, 1987). For example, finding the ‘best’ clustering to identify new disease subtypes	- Usually handles outliers better than DBI (Dixon et al., 2009) - Useful for identifying the optimal number of clusters (Shahapure and Nicholas, 2020)	- Cannot detect if the clustering is due to a bias in the data that is unrelated to the trait (Chhabra et al., 2021) - Assumptions rely on Gaussian clusters so unsuitable for rare disease clusters or sparse data (Thrun, 2018)	- Clustering Multiple Sclerosis (MS) patients based on GWAS data (Lopez et al., 2018) - Clustering schizophrenia patients based on clinical and genetic data (Yin et al., 2018)
Davies-Bouldin Index (DBI)	Compares the similarity between each cluster and the cluster most similar to it (Davies and Bouldin, 1979). For example, finding the ‘best’ clustering to identify new disease subtypes	- Simpler and more efficient computation than SI (Petrovi´c, 2006) - Handles different shapes and cluster count better than SI and CHI (Davies and Bouldin, 1979)	- Requires Euclidean distances which are not always suitable, e.g., in sparse datasets (Davies and Bouldin, 1979) - Cannot compare between datasets (Dixon et al., 2009)	- Gene expression clustering for systematic autoinflammatory diseases (Papagiannopoulos et al., 2024) - Clustering single-cell transcriptomes for identification of cell types and states (Zhao et al., 2023)
Calsinki-Harabasz Index (CHI)	Compares the similarity within clusters to the distance from the cluster to the global centre (Caliñski and Harabasz, 1974). For example, finding the ‘best’ clustering to identify new disease subtypes	- Simple and efficient computation, an important consideration for large genomics datasets (Caliñski and Harabasz, 1974)	- Assumes that clusters have equal size and density (Caliñski and Harabasz, 1974). Spherical assumptions are unsuitable for imbalanced clusters (e.g., rare disease clusters)	- Risk stratification from electronic health record data (Huang et al., 2021) - Gene clustering from single-cell data with reduced uncertainty (Li et al., 2023)
Gap Statistics	Compares within cluster variation to the expected value from a reference distribution (Tibshirani et al., 2001). A method for selecting the optimal number of clusters but can also be used as a metric with higher values indicating it is significantly better than random	- Useful for identifying optimal cluster numbers (Tibshirani et al., 2001) - Useful for evaluating the clusters with respect to random noise (Tibshirani et al., 2001). This is helpful in genomics where there is uncertainty over whether the disease or variants being clustered have subtypes or not	- Not as direct as the previously listed metrics - Relies on comparison with random distribution, not comparing clustering properties (Tibshirani et al., 2001)	- Clustering type 2 diabetes based on clinical biomarkers (Lugner et al., 2021) - Choosing the number of clusters for population clustering based on short tandem repeats (STRs) (Syukriani and Hidayat, 2023)
Accuracy	Percentage of samples correctly predicted. For example, the percentage of individuals correctly labelled diseased or control	- Very simple to understand	- Heavily impacted by imbalanced datasets which are common in genomics (Bone et al., 2015; Poldrack et al., 2020)	- Prediction of schizophrenia from genetic and clinical data on comorbid conditions (Chen et al., 2018) - Prediction of ADHD from genetic variants (Liu et al., 2021)
Precision	Percentage of samples predicted to be “positive” that are actually “positive”. For example, the percentage of identified variants that are predicted correctly	- Useful when false positives are more detrimental than false negatives	- Only considers the positive predictions (e.g., predicted cases)	- Identifying drug sensitive cancer cell lines (Naulaerts et al., 2017) - Analysing gene expression profiles from microarray data while maintaining high precision (Salem et al., 2017)
Recall	Percentage of “positive” samples that were correctly predicted. For example, the percentage of breast cancer cases correctly predicted	- Useful when false negatives are more detrimental than false positives	- Only considers the positive class (e.g., cases). You could get 100% recall by predicting everyone to be a case	- Improving recall of taxonomic metagenomic sequence classification (Girotto et al., 2017) - Early detection of cervical cancer with high recall (Gupta et al., 2021)
F1	The harmonic mean of precision and recall. For example, minimising both missed diagnoses (false negatives) and incorrect diagnoses (false positives) in a genetic testing algorithm	- Focusses on the trade-off between precision and recall in one metric - More suitable for imbalanced data than accuracy, however, less so than AUROC (Jeni et al., 2013)	- Does not consider true negatives which can be important (e.g., identifying individuals who do not carry a specific mutation in carrier screening)	- Training geneformer, a model using single-cell transcriptomes for context aware predictions of, e.g., gene network dynamic (Theodoris et al., 2023) - Survival prediction of heptocelluar cancer based on clinical data and biomarkers (Książek et al., 2021)
Area Under Receiver-Operator Curve (AUROC)	The area under the curve (AUC) of the true positive rate (TPR) plotted against the false positive rate (FPR). Often used to compare different ML models for predicting a certain disease or variant types	- Useful in an objective model comparison, particularly when the optimal decision boundary is unknown - Visualises the trade-off between TPR and FPR.	- Alone it provides little clinical significance as it is not at a specific decision boundary - Susceptible to biases from imbalanced and small datasets which are common in genomics (however, less so than accuracy) (Faviez et al., 2020) - Gives false positives and false negatives the same weighting; often not the case in genomics (Ioannidis et al., 2011)	- Prediction of Parkinson’s disease from genetic variants (Ho et al., 2022) - Prediction of Alzheimer’s disease from gene expression data (Lee and Lee, 2020)
Matthews Correlation Coefficient (MCC)	A balanced metric to evaluate classification predictions considering true negatives (TN), true positives (TP), false negatives (FN), and false positives (FP) (Matthews, 1975; Baldi et al., 2000)	- Considers all confusion matrix components (TN, TP, FN, FP) - Handles imbalanced data better than accuracy, F1 and AUROC (Chicco and Jurman, 2020; 2023)	- Currently less known so less familiar to readers without a ML background	- Predicting melanoma from mRNA and methylation data (Bhalla et al., 2019) - Predicting cancer progression from RNAseq data (Singh et al., 2018)
Cohen’s kappa	Evaluates the level of agreement between two groups (originally between two raters, now often between predictions and ground truth) taking into account chance agreement (Ben-David, 2008)	- Accounts for agreement expected by chance (Ben-David, 2008)	- Less intuitive to set a threshold in clinical settings as it is a relative measure - Not robust to asymmetric confusion matrices or imbalanced data and can therefore give conflicting values to MCC (Jeni et al., 2013; Delgado and Tibau, 2019)	- Microbial risk assessment using next-generation sequencing (NGS) (Njage et al., 2019) - Predicting individuals’ lithium response from genetic variants (Stone et al., 2021)
Mean Absolute Error (MAE)	The average absolute difference between the predicted values and known values. For example, the average distance (in kg) that a model is from predicting birth weight	- Easy to interpret as shares units with measurements - Low sensitivity to outliers (Hodson, 2022)	- Cannot be used to compare the predictions of datasets with different variances	- Predicting bone mineral density form genetic variants (Wu et al., 2021) - Predicting gene expression from ‘landmark genes’ using cluster-based regression (Seok, 2021)
Root Mean Squared Error (RMSE)	Similar to MAE, it is the average absolute difference between the predicted values and known values. However, it is the square root of the mean squared error	- Easy to interpret as shares units with measurements	- Higher outlier sensitivity than MAE (Hodson, 2022)	- Predicting BMI from clinical and genetic data (Harrison et al., 2017) - Analysing association between body fat and cardiovascular risk (Saito et al., 2017)
R-squared Error (R^2^)	Proportion of variation in the target variable that the regression model explains. For example, the percentage of variation in height explained by a regression model with known biomarkers	- Unitless so easy to compare different models	- Relying on a high R^2^ during model tuning can lead to overfitting (Bohrnstedt and Carter, 1971) - Tends to increase as parameters added (fixed with adjusted R^2^) (Bohrnstedt and Carter, 1971)	- Analysing association between genetic scores and birth weight. Used R^2^ and adjusted R^2^ (Haulder et al., 2022) - Comparing predictability of genetic risk scores for different traits across different ancestral groups (Ekoru et al., 2021)

## 3 ML metrics for classification

Classification is the machine learning category most frequently used in genetics and genomics (Al-Tashi et al., 2023; Ho et al., 2022; Lee and Lee, 2020; Trakadis et al., 2019). Whilst the classification method complexity can range from simple logistic regression to complicated deep learning algorithms, the metrics remain predominantly the same. For parametric classifiers, the choice of metric largely depends on (1) the distribution of the data and (2) an understanding of the aim of the study. Nonparametric decision boundaries do not make assumptions about the data’s distribution (e.g., DD-classifier (Li et al., 2012)), however, these are not covered in this paper. Common metrics include: accuracy, area under the receiver-operator curve (AUROC), precision, recall, and F1.

### 3.1 Accuracy

Accuracy is the simplest classification metric to understand and is often reported in genomics papers (Chen et al., 2018; Trakadis et al., 2019; Liu et al., 2021). Accuracy provides a measure of the percentage of individuals who are correctly classified.

$$Accuracy=\frac{no.\;of\;correct\;classifications}{total\;no.\;of\;classifications}\times 100$$

The accuracy metric is used to evaluate how well an algorithm assigns individuals to the correct category (e.g., predicting whether someone has a particular trait or not). However, accuracy is heavily impacted by imbalanced datasets (Bone et al., 2015; Poldrack et al., 2020). For example, if a dataset of 100 individuals contains 10 diseased individuals and 90 healthy, an algorithm could get an accuracy of 90% by predicting everyone to be healthy. This is a real issue for genomic analyses, as they are often imbalanced due to the ease of obtaining data from control in comparison to the affected individuals, especially when dealing with rare traits/diseases (Devarriya et al., 2020; Faviez et al., 2020; Dai et al., 2021). Therefore, it is important to understand the dataset structure to enable an objective assessment of the accuracy measure.

### 3.2 Precision, recall, and F1

**FIGURE 3:**
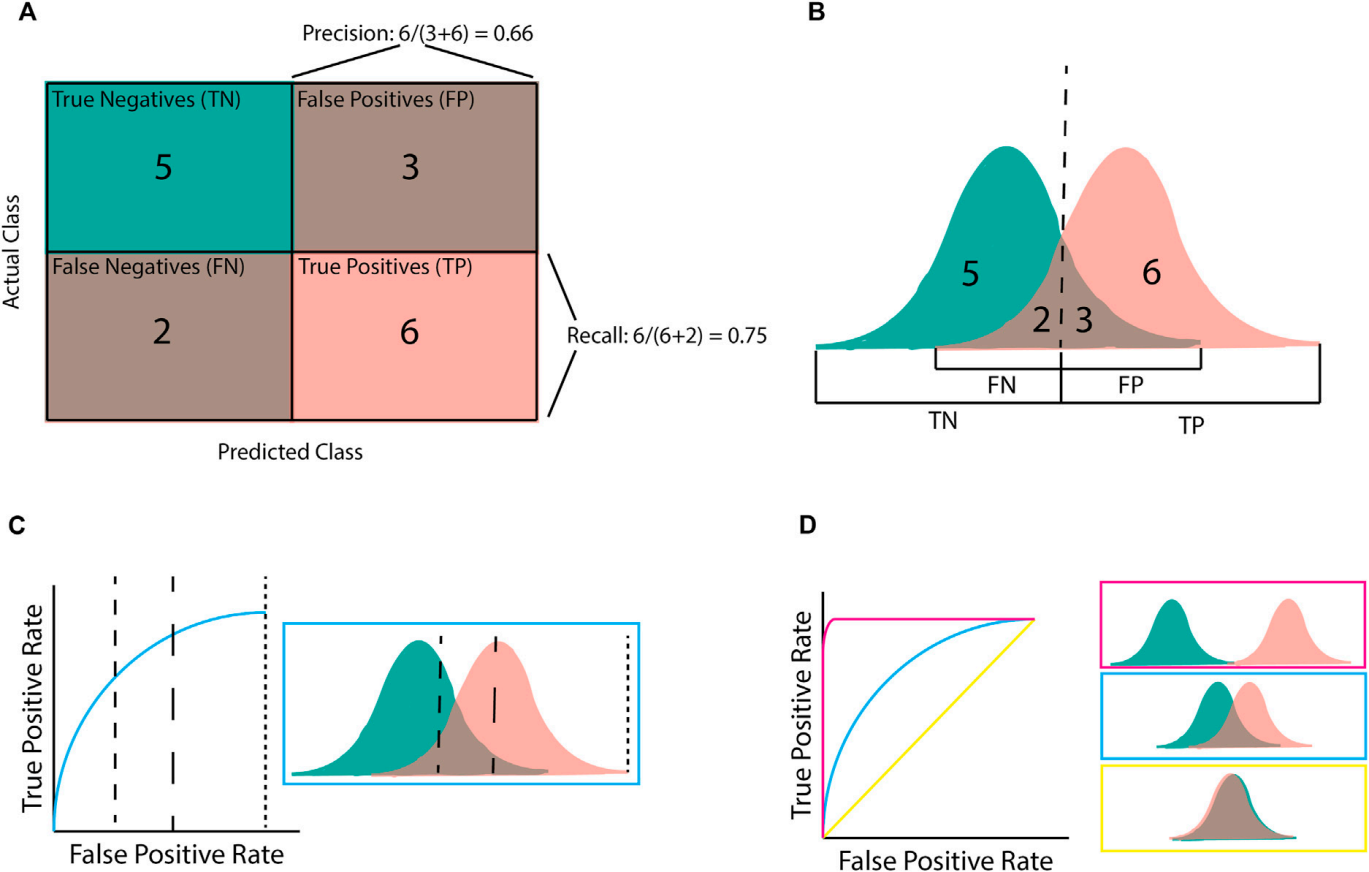
Illustration of classification metrics. **(A)** Confusion matrix used to calculate precision and recall. **(B)** the score distribution and threshold that gives the confusion matrix in **(A)**. Every score below the dashed line is assigned to the negative class whilst scores after the dashed line are assigned to the positive class. **(C)** An Area Under the Receiver-Operator Curve (AUROC) graph for the given score distribution. Different chosen thresholds (dashed lines) give different ratios of FPR to TPR. **(D)** AUROC graphs for the three distribution patterns. Pink shows complete separation, blue is partial separation, and yellow is complete crossover.

Confusion matrices (Figure 3A) are a simple way to display predictions for a population by separating them into those that were correctly predicted to be controls (true negatives; TN), correctly predicted to be cases (true positives; TP), incorrectly predicted to be controls (false negatives; FN), and incorrectly predicted to be cases (false positives; FP) (Figures 3A, B). The precision, recall, and F1 classification scores can be calculated from these four groups.

$$Precision=\frac{TP}{TP+FP}\;Recall=\frac{TP}{TP+FN}\;F1=\frac{2\times P\times R}{P+R}$$

Given:

- TP: number of true positives
- FP: number of false positives
- FN: number of false negatives
- P: precision
- R: recall

Precision (or positive predictive value [PPV]) refers to the percentage of samples predicted to be “positive” that are actually “positive”; that is, a 100% precision means that there were no samples incorrectly labelled as “positive”. However, precision does not consider positive samples that were incorrectly labelled “negative”. By contrast, recall (or sensitivity) refers to the percentage of “positive” samples that were correctly labelled as positive; that is, a 100% recall score means that no positive samples were incorrectly labelled as negative. The F1 score is the harmonic mean of these precision and recall metrics. Therefore, a high F1 score requires both a high precision and a high recall. The importance of the precision and recall metrics varies according to the problem. For example, if an algorithm has been designed for disease diagnosis, incorrectly labelling an individual as health would be more harmful, making recall more important than precision (Chen et al., 2017). On the contrary, if an algorithm focuses on identifying genetic variants of interest or transcriptional effects, it is more important that the majority of the identified variants are correctly predicted, even at the expense of missed variants (false negatives). In this case precision would be more important than recall (Ioannidis et al., 2011).

### 3.3 Area under a receiver-operator curve

Area under a Receiver-Operator Curve (AUROC) is a common metric used in genomics as it is helpful for model comparison (Lee and Lee, 2020; Gupta et al., 2022; Ho et al., 2022). AUROC is calculated by plotting the true positive rate (TPR; equivalent to recall) against the false positive rate (FPR) and finding the area underneath this curve (Figures 3C, D). AUROC quantifies how well a model distinguishes between different classes by summarising the model’s performance at all decision boundaries (Box 1) into one value. This is different from other metrics (e.g., accuracy, precision, and recall) that only consider the model at a given decision boundary. However, even though AUROC is commonly used in genomics, it is not always useful on its own as, despite being helpful for model comparison, using AUROC alone provides little measure of clinical significance. For example, AUROC does not provide insight into how well a specific model will perform upon deployment (e.g., for diagnosing a disease) as this requires a decision boundary to have been chosen and validated.

Two keys assumptions limit the use of AUROC. Firstly, AUROC assumes false positives and false negatives are equally undesirable, which is not always the case in genomic analyses where the consequences of incorrectly predicting someone has not got a particular condition (false negative) can be far greater than the consequences of incorrectly predicting that they do (false positives) (Ioannidis et al., 2011). Secondly, AUROC is susceptible to biases from imbalanced and small datasets, both of which are common in genomics, particularly within studies of rare diseases (Faviez et al., 2020). Given these limitations, many studies will report the AUROC metric alongside metrics that include accuracy, precision, and recall, which are calculated at a given decision boundary and thus provide more clinical significance (Gao et al., 2021; Liu et al., 2021).

### 3.4 Matthew’s correlation coefficient and Cohen’s kappa

The above metrics are a selection of those most commonly used in ML for genomics and are arguably the easiest to understand. However, like with clustering, there are many other metrics available. Two metrics that are increasing in popularity and address some of the disadvantages of the metrics listed above are Matthew’s correlation coefficient (MCC) (Singh et al., 2018; Bhalla et al., 2019; Chicco and Jurman, 2020) and Cohen’s kappa (Ben-David, 2008; Njage et al., 2019; Yu et al., 2019; Stone et al., 2021). Particularly, MCC has been suggested as a preferential metric to the more popular ones discussed in this section due to its increased reliability with imbalanced datasets (Chicco and Jurman, 2020; 2023). Advantages, disadvantages, and use cases for these are listed in Table 1.

## 4 ML metrics for regression

Regression is less common in genomic studies than classification. However, it is helpful in predicting highly heterogenous traits with known scales such as height, systolic blood pressure, and waist-hip ratio (Bellot et al., 2018; Lello et al., 2018). The choice of regression metric for a particular analysis is also more nuanced than in classification studies, as the advantages and disadvantages of each option are less obvious. However, regression metrics that are commonly used include mean absolute error (Shahid and Singh, 2020), root mean squared error (Shmoish et al., 2021), and R^2^ (Harrison et al., 2017; Haulder et al., 2022).

### 4.1 Mean absolute error

Mean absolute error (MAE) is a common method for measuring the average difference between the predicted values and the known values.

$$MAE=\sum_{i=1}^{n}\frac{\left|y_{i}-x_{i}\right|}{n}$$

Given:

- $x_{i}$ = predicted value i

- $y_{i}$ = true value i

- n = number of data points

The units for MAE are the same as the data points, making it easier to understand. However, this means it is hard to compare different predictions if the underlying data have different units. For example, Lello et al. (2018) used machine learning to predict height, heel bone density, and educational attainment from the same dataset (UK Biobank). They chose to look at the total variance explained by the model, however, had they chosen MAE as their metric instead, they would not be able to easily compare the predictability of the three traits – due to the different units used to measure each trait.

MAE has several strengths that make it useful, in particular MAE is less sensitive to outliers as it gives equal weight to all errors (Hodson, 2022). However, giving equal weighting to all errors means MAE cannot be used to compare the predictions of datasets with different variances even when these incorporate the same measurement units (e.g., predicting two body measurements in datasets with differing variance).

To take advantage of the strengths and restrict the impact of the limitations associated with the use of MAE, many researchers choose to report MAE alongside other metrics, such as root mean squared error and R^2^ (see below) (Shahid and Singh, 2020; Shmoish et al., 2021; Zhang et al., 2021).

### 4.2 Root mean squared error

Root mean squared error (RMSE) is another frequently used metric for measuring the average difference between the predicted values and actual values in regression.

$$RMSE=\sqrt{\frac{\sum_{i=1}^{n}{\left(y_{i}-x_{i}\right)}^{2}}{n}}$$

Given:

- $x_{i}$ = predicted value i

- $y_{i}$ = true value i

- n = number of data points

Similar to MAE, the units for RMSE are the same as those used for the data points. However, RMSE is more sensitive to outliers than MAE. This means RMSE gives larger weightings to these errors (Hodson, 2022). As such, whether MAE or RMSE is a better error metric has been hotly debated. Willmott et al. (2009) argued that sums-of-square-based statistics such as RMSE can not be used to represent average error as they vary in response to both error variability and central location. Chai and Draxler (2014) debated this, using simulations to show that RMSE is not only not ambiguous, but is more valuable than the MAE when the expected error distribution is Gaussian. It has also been suggested that a ratio of the two metrics is a more accurate metric than either option individually (Karunasingha, 2022).

RMSE has been used in genomic studies as a metric for predicting heterogeneous traits (Harrison et al., 2017; Shmoish et al., 2021). However, like MAE, RMSE is typically reported alongside the R^2^ error, which measures the proportion of variation explained by the model (see below) (Harrison et al., 2017; Shmoish et al., 2021).

### 4.3 R-squared error

The R-squared error (R^2^), also known as the coefficient of determination, provides a measure of the proportion of variation in the variable being predicted (target variable) that the regression model explains.

$$R^{2}=1-\frac{\sum_{i=1}^{n}{\left(y_{i}-x_{i}\right)}^{2}}{\sum_{i=1}^{n}{\left(y_{i}-y_{mean}\right)}^{2}}$$

Given:

- $x_{i}$ = predicted value i

- $y_{i}$ = true value i

- $y_{mean}$ = mean of true values

- n = number of data points

Unlike MAE and RMSE, R^2^ error is not measured in the same units as the data points but instead varies from 0 (model explains 0% of target variable variance) to 1 (model explains 100% of target variable variance). Because of this, R^2^ error is easily used to compare different models. A large R^2^ suggests that the model is a good fit for the data. On the other hand, low R^2^ values can mean that there is a significant amount of noise compared to signal (i.e., low signal-to-noise ratio). A low R^2^ is not always bad, however, as it may just be indicative of low effect sizes which are common in complex disease genetics (Marian, 2012). Conversely, a high R^2^ is not always good. Relying on a high R^2^ for model tuning can result in overfitting as it is not robust to the number of predictors (Bohrnstedt and Carter, 1971). Notably, R^2^ tends to increase when new variables are added to the model, even if they do not cause significant improvement(s) (Bohrnstedt and Carter, 1971). This can be compensated for by using the adjusted R^2^.

$$Adjusted\;R^{2}=1-\frac{\left(1-R^{2}\right)\left(n-1\right)}{n-p-1}$$

Given:

- n = number of data points
- p = number of independent variables/predictors

The adjusted R^2^ decreases if the additional parameters do not increase the model’s predictability. Therefore, the adjusted R^2^ is often a more suitable measure for genomic studies, where models frequently use many variables (e.g., many genes, clinical scores, sex, and anthropometric measures) to predict target variables (e.g., birthweight) (Haulder et al., 2022).

## 5 Common pitfalls that lead to exaggerated metrics

Regardless of the chosen metric, some common pitfalls can result in the wrong conclusions being drawn. This can be particularly problematic in genetic and genomic studies, especially if a published model is thought to be more accurate at predicting a disease than it is. However, overfitting of data is the main cause of exaggerated metrics (England and Cheng, 2019). A model is considered overfit if it predicts extremely well for the training data but is a poor predictor outside the context. The chance of overfitting is greatly reduced by splitting the data into a training and test dataset, however, if enough models are trained on the training dataset, it is possible to find one that performs well on the test dataset by chance. For example, Chekroud et al. (2024) found that a machine learning model designed to predict patient outcomes of individuals in schizophrenia drug trials had high accuracy for predictions within the trial dataset used to train the model. However, in other trials its performance was no better than chance (Chekroud et al., 2024). Therefore, when optimising a model to achieve higher scores in the chosen metrics, it is crucial to remember that the scores are only relevant for the dataset(s) that the model is trained and tested on. This relates to the concept “bias-variance tradeoff” where high bias comes from a simplified model and leads to underfitting whereas high variance comes from a complex model with low training errors, leading to overfitting (Geman et al., 1992). As mentioned in the previous section, some metrics (including R^2^) are more prone to overfitting, and adjustments can be made to minimise this problem (e.g., adjusted R^2^). Reproducibility is critical so that the pipeline can be repeated on another dataset to confirm the validity of the model’s claims (Pineau et al., 2021) and identify overfitting.

Another common cause of exaggerated metrics is if the test data does not remain unseen by the model during training. That is, the test data must be kept hidden throughout feature selection and model training. Otherwise the model may learn features from the test dataset that it would not have otherwise learnt. A common mistake is to split the data after feature selection has begun (e.g., after genes or SNPs have been selected based on a statistical test), however, doing so will lead to inflated metrics (Kapoor and Narayanan, 2023). For example, Barnett et al. (2023) found that 44% of the genomic studies they investigated had inflated metrics due to data leakage during feature selection. On average, they saw an AUROC increase of 0.18 because of this data leakage. Unlike with overfitting, all metrics are equally impacted by this bias so care must be taken both during model training and when evaluating the metric scores. Again, reproducibility is essential to confirm the validity of the model’s claims and identify any biases.

Even if an effort is made to ensure the data is not overfit to the training data and the test data remains unseen, it is important to understand the limitations of the dataset. Models created with data from a specific subpopulation may not be meaningful when applied to other populations (De Roos et al., 2009; Gurdasani et al., 2019). For example, an algorithm using SNP information within a European population to predict a disease may not be as accurate when applied to different population groups. Understanding the dataset means it is easier to check for any biases inflating the reported metrics. For a dataset of individuals with and without a particular disease, if there is information on ancestry or sex, a simple check should be performed to confirm that the model remains unbiased toward a specific group. If there is a disparity in metric scores between groups, reporting the metrics for the different groups separately brings awareness to these biases.

A checklist of standards for publishing papers on AI-based science has been created that covers eight sections, including metrics and reproducibility (Kapoor et al., 2023). Specific reproducibility standards for the life sciences have also been published (Heil et al., 2021).

## 6 Discussion

Machine learning is a powerful tool within genetic and genomic research and has become increasingly accessible to researchers. However, care must be taken when choosing a metric for evaluating model outputs and interpreting the results. There is no one-size-fits-all metric available. We contend that multiple suitable performance metrics should be chosen based on an understanding of the dataset and the research question. Result reproducibility is crucial for readers to trust the reported metrics, as is a discussion of potential biases within the data and model that could have impacted the metrics. After reporting on the model’s performance, biases should be considered. It is best to keep the research question and data context in mind throughout the process to ensure reliable and confident results.
